# The Immune System through the Lens of Alcohol Intake and Gut Microbiota

**DOI:** 10.3390/ijms22147485

**Published:** 2021-07-13

**Authors:** Javier Calleja-Conde, Victor Echeverry-Alzate, Kora-Mareen Bühler, Pedro Durán-González, Jose Ángel Morales-García, Lucía Segovia-Rodríguez, Fernando Rodríguez de Fonseca, Elena Giné, Jose Antonio López-Moreno

**Affiliations:** 1 Departamento de Psicobiología y Metodología en Ciencias del Comportamiento, Facultad de Psicología, Universidad Complutense de Madrid, 28223 Madrid, Spain; jcconde@ucm.es (J.C.-C.); echeverry.v@psi.ucm.es (V.E.-A.); kobuhler@psi.ucm.es (K.-M.B.); pduran01@ucm.es (P.D.-G.); luciaseg@ucm.es (L.S.-R.); 2 Unidad Gestión Clínica de Salud Mental, Instituto de Investigación Biomédica de Málaga (IBIMA), Hospital Regional Universitario de Málaga, Malaga University, 29010 Málaga, Spain; fernando.rodriguez@ibima.eu; 3 Universidad Nebrija, Campus Madrid-Princesa, 28015 Madrid, Spain; 4 Instituto de Investigaciones Biomédicas (CSIC-UAM) “Alberto Sols” (CSIC-UAM), 28029 Madrid, Spain; jmoral06@ucm.es; 5 Departamento de Biología Celular, Facultad de Medicina, Universidad Complutense de Madrid, 28040 Madrid, Spain; elena.gine@med.ucm.es; 6 Centro de Investigación Biomédica en Red en Enfermedades Neurodegenerativas (CIBERNED), 28031 Madrid, Spain

**Keywords:** alcohol, gut, microbiota, brain, liver, immune system, dysbiosis

## Abstract

The human gut is the largest organ with immune function in our body, responsible for regulating the homeostasis of the intestinal barrier. A diverse, complex and dynamic population of microorganisms, called microbiota, which exert a significant impact on the host during homeostasis and disease, supports this role. In fact, intestinal bacteria maintain immune and metabolic homeostasis, protecting our organism against pathogens. The development of numerous inflammatory disorders and infections has been linked to altered gut bacterial composition or dysbiosis. Multiple factors contribute to the establishment of the human gut microbiota. For instance, diet is considered as one of the many drivers in shaping the gut microbiota across the lifetime. By contrast, alcohol is one of the many factors that disrupt the proper functioning of the gut, leading to a disruption of the intestinal barrier integrity that increases the permeability of the mucosa, with the final result of a disrupted mucosal immunity. This damage to the permeability of the intestinal membrane allows bacteria and their components to enter the blood tissue, reaching other organs such as the liver or the brain. Although chronic heavy drinking has harmful effects on the immune system cells at the systemic level, this review focuses on the effect produced on gut, brain and liver, because of their significance in the link between alcohol consumption, gut microbiota and the immune system.

## 1. Gut Microbiota and Immune System

The human body contains many different types of cells. These cells include both human cells (mainly erythrocytes) and non-human cells such as bacteria, fungi, yeasts and viruses. In fact, given a standard 70 kg human male, there are slightly more bacteria than human cells, being the estimated bacteria/human ratio 1:3 [1]. This collection of microbes that inhabit a human body represent the human microbiota. 

In the human body, the gut represents the organ with the largest surface area (approximately 32 m^2^) [2] as well as the one with the highest number of microbes, especially in the colon, where the density of bacterial cells has been estimated at 10^11^ to 10^12^ per milliliter [3]. After a child reaches the age of three, the bacterial composition of gut microbiota remains reasonably stable and is unique to everyone depending on different factors like genetics, diet, and different environmental factors. A healthy gut microbiota is characterized by its richness and diversity in its composition [4]. Nevertheless, studies have shown that the normal gut microbiota comprises mainly *Bacteroidetes* and *Firmicutes* as the dominant phyla, followed by *Actinobacteria* and *Verrucomicrobia*. These gut commensals play an important role in specific functions like nutrient and drug metabolism, protection against pathogens, maintenance of structural integrity of gut mucosal barrier, among others [5,6].

During the last years, a substantial number of studies on the impact of gut microbiota and host health have been conducted, showing that the disruption of gut microbiota homeostasis (called dysbiosis) is related to a large array of diseases. These include metabolic diseases like obesity and metabolic-associated fatty liver disease [7,8], irritable bowel syndrome [9] as well as several immune-related diseases like allergies [10], autoimmune diseases [11], and inflammatory bowel disease [12]. These latter associations illustrate the direct interaction between gut microbiota and the immune system. 

**Figure 1:**
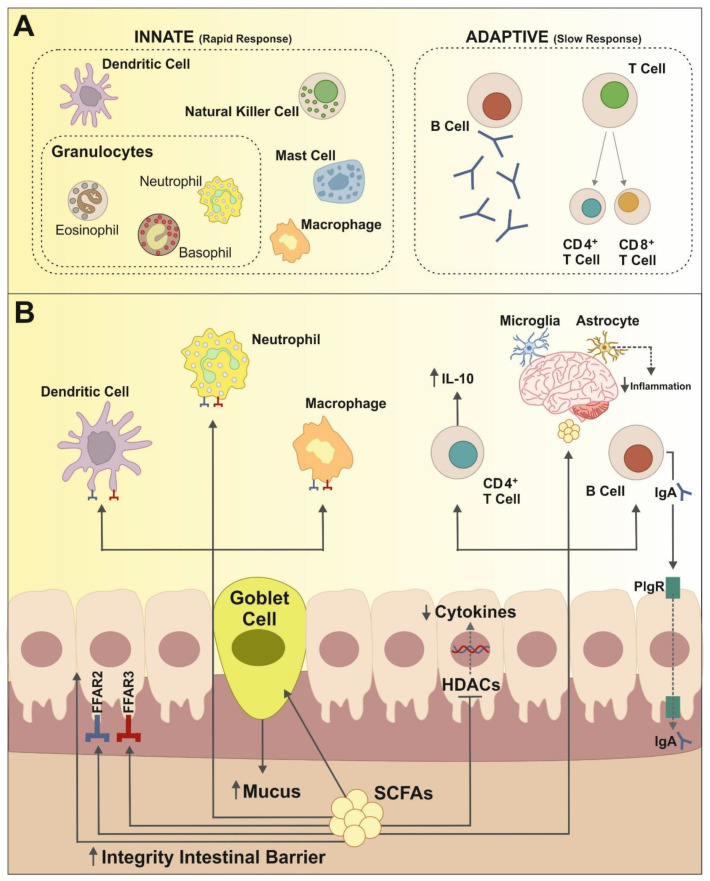
Relationships between the innate and the adaptive immune systems and gut microbiota. (**A**) The innate immune response is a very fast, pathogen-non-specific, first line of defense mechanism. It is mainly composed of macrophages, dendritic and natural killer cells, as well as different forms of granulocytes. The adaptive immune system is highly specific to a particular pathogen and is formed by B and T cells lymphocytes. (**B**) The gut microbiota is in close interaction with both the innate and the adaptive immune system. This interaction is frequently driven by SCFAs, which modulate local as well as systemic immune response. SCFAs can bind to G-protein-coupled receptors as FFAR2 and FFAR3 present on the surface of gut epithelial cells and immune cells including dendritic cells, macrophages and neutrophils, and are therefore important regulators of inflammatory response. SCFAs also promote the activation of B cells and the development of Treg CD4+T cells—for example, increasing secretion of IL-10 with important anti-inflammatory effects. Suppression of inflammatory factors like cytokines is further achieved by the inhibition of histone deacetylases (HDACs) activity. Finally, SCFAs have been shown to modulate immune inflammation responses in extraintestinal organs such as the brain, by acting on microglia and astrocytes.

Mounting evidence based on humans and rodent models, mainly germ-free mice, support the idea that the gut microbiota is in constant crosstalk with the immune system [13,14]. The immune system is a complex network that includes molecules, cells, tissues, and organs that defend the body against infectious agents and malignant cells. It is broadly divided into innate and adaptive immune systems. The innate immunity response is not specific for any pathogen, and its cells (i.e., natural killer cells, neutrophils, monocytes/macrophages and dendritic cells) express pathogen recognition receptors (PRRs), such as Toll-like receptors (TLRs), that recognize pathogen-associated molecular patterns (PAMPs) that are part of many microorganisms but not of the host body’s own cells (Figure 1A). On the other hand, the adaptive immune system can be subdivided into cell-mediated immunity, carried out by T cells, and humoral immunity (B-cells responses). T cells expressing the CD4 T cell co-receptor, called T helper cells, are involved in the activation and maturation of monocytes, cytotoxic T cells (expressing the CD8 T cell co-receptor) and B cells. Cytotoxic T cells eliminate cancer cells and intracellular pathogens. B cells mature into plasma cells that produce antibodies (immunoglobulins, Ig), eliminating extracellular microorganisms and preventing the spread of infection [15,16] (Figure 1A). Both the innate and the adaptive immune system influences and shapes the composition and diversity of the gut microbiota [17], whereas the gut microbiota modulates the immune system via different metabolites and activation of TLRs signaling pathways [18]. 

Maintaining gut homeostasis—beneficial microbiota composition—plays a critical role in immune responses. By fermentation of complex carbohydrates, anaerobic bacteria in the gut produce short-chain-fatty acids (SCFAs), which are essential for modulation and mediation of the immune system. SCFAs produced in the gut are mainly butyrate, propionate and acetate and have many different targets and functions in the host organism. SCFAs regulate local immune response in the gut, as well as they act as important immune mediators in extra-intestinal organs such as the brain and the liver as well as in other tissues (for example, skin, lungs and pancreas) [19].

In the gut, the uptake of SCFAs by intestinal epithelial cells (IECs), mainly butyrate, promotes the integrity of the intestinal barrier, reducing intestinal permeability [20] and, therefore, preventing bacterial translocation through the gut wall and the resulting endotoxemia and associated immune response [17]. SCFAs also exhibits important anti-inflammatory effects on gut immune cells. For example, butyrate stimulated differentiation of T-regulatory cells and increased levels of IL-10 while reducing production of IL-6 and inhibiting the expansion of pro-inflammatory Th17 cells [21]. Moreover, SCFAs show epigenetic regulatory effects by inhibiting HDACS promoting in this way the suppression of inflammatory responses in immune cells [22,23] as well as promoting production of IgA and IgG antibodies by B cells. SCFAs have also shown to be natural ligands for free fatty acid receptor 2 and 3 (FFAR 2 and FFAR 3 also known as GPR43 and GPR41, respectively) [24]. In particular, FFAR2 are highly related to immune cell function and mast cell activity since they are expressed in neutrophils, macrophages and dendritic cells, among others (Figure 1B). Activation of FFAR2 has been associated with the maintenance of gut homeostasis and regulation of inflammation related to disease such as asthma, allergies, cardiovascular and fatty-liver disease [25].

SCFAs have been associated with normal development of brain resident immune cells, specifically with microglia and astrocytes. In the brain, microglia are the most abundant immune cells and perform a variety of functions including phagocytosis, cytokine production and activation of inflammatory response, between others [26]. As observed in germ-free mice as well as in animals presenting FFAR2 abnormalities, alterations in gut microbiota lead to abnormal microglial abundance, morphology, and gene expression patterns [27,28]. Astrocytes, on the other hand, are the most frequent glial cells in the brain and perform several immune related functions including the expression of pattern recognition receptors for detection of microbial-associated molecular pattern (MAMPs) and modulation of the neuroinflammatory response [29]. Metabolites produced in the gut by metabolization of dietary tryptophan are able to bind to astrocyte aryl hydrocarbon receptors (AHR) reducing by this way proinflammatory factors (Figure 1B). Therefore, intestinal bacteria seem to be an important regulator of neuroinflammation. This idea has been supported by different studies using a mouse model of multiple sclerosis (called experimental autoimmune encephalomyelitis, EAE) showing a protective effect of SCFAs by increasing IL-10 producing regulatory T cells differentiation. Altogether, this interaction between gut microbiota and immune system on the gut-brain axis plays an important role in the etiopathogenesis of psychiatric and neurological diseases such as autism spectrum disorder, depression and addiction, among others [13,30].

The interaction between the liver immune system and the microbiome, under normal health conditions, is limited. Only select substances can cross the intestinal barrier and move into the liver, the bile ducts and the portal vein being the major connection points between the liver and microbiome [31]. However, in certain contexts, when intestinal commensals and their products translocate from the intestinal lumen to the liver, hepatic immune responses may be affected [32]. For example, the number, functional activity, and maturational status of the hepatic Kupffer cells (KCs), a critical component of the hepatic innate immune system, are directly related to the concentration of gut-derived MAMPs [33]. Intestinal pathogenic bacteria facilitate immune-mediated liver injury by activating dendritic cells (DCs) and natural killer T (NKT) cells in the liver [34]. Additionally, it has been reported that probiotics may contain bacterial glycolipid antigens that stimulate hepatic NKT cells in a strain-specific and dose dependent manner [35].

## 2. Effects of Alcohol on Gut Microbiota

Alcohol addiction is a leading risk factor for personal death and disability. In 2016, the harmful use of alcohol resulted in some 3 million deaths (5.3% of all deaths) worldwide and 132.6 million disability-adjusted life years (DALYs), i.e., 5.1% of all DALYs in that year. Among men in 2016, an estimated 2.3 million deaths and 106.5 million DALYs were attributable to the consumption of alcohol. Women experienced 0.7 million deaths and 26.1 million DALYs attributable to alcohol consumption [36].

Alcohol abuse represents a risk factor for liver diseases, such as alcoholic steatohepatitis and cirrhosis [37] in such a way that approximately 25% of heavy drinkers develop clinically alcoholic liver disease (ALD).

Although alcohol is absorbed through the mucosa of the entirely gastrointestinal tract by simple diffusion, it is mainly absorbed in the upper part of the tract [38], the majority of it (70%) in the small intestine [39]. The large part of alcohol metabolism in humans occurs in the hepatocytes, main cells of the liver. Ethanol is metabolized by alcohol dehydrogenases (ADH), catalase or cytochrome P450 2E1 to acetaldehyde which is then further oxidized to acetate by aldehyde dehydrogenase (ALDH) [40]. Ninety percent of the moderate alcohol consumed is metabolized through oxidative conversion by alcohol dehydrogenases enzymes while the microsomal ethanol–oxidizing system (MEOS) handles the remaining 10%; this last route acquires greater importance when alcohol consumption increases significantly. MEOS leads to the production of oxygen free radicals, which can cause cellular damage [41]. Besides in the liver, the enzymes involved in the oxidative metabolism of alcohol also are present in the intestinal mucosa and intestinal bacteria also produce acetaldehyde in the gastrointestinal tract [41].

The intestinal microbiota (IMB) is the set of microorganisms that inhabit our intestines. These microorganisms, among others, include bacteria, fungi, yeasts and viruses [42]. However, in most cases, when referring to IMB, one usually refers to the populations of bacteria that have colonized our large intestine. Gut dysbiosis, which may result in an overgrowth of Gram-negative bacteria [38], can be yielded by the direct toxicity of the alcohol or by indirect mechanisms triggered by alcohol such as the alteration of gut motility [43], the gastric acid output [44], the bile-acid metabolism [45] and an increase in fecal pH [46].

To date, most studies have reported that heavy alcohol consumption directly alters the biodiversity of gut microbes and produces dramatic change in the relative abundance of some particular microbes, causing dysbiosis and inflammation in the gut [47,48,49]. Similar effects have been shown in moderate alcohol consumption and chronic consumption in animal models [46,50,51,52]. Intestinal dysbiosis was correlated with the amount of alcohol consumed [47]. Although the changes are specific to the species studied (rodents or humans) and the alcohol ingestion protocol, there is trend for a depletion of bacteria with anti-inflammatory activity, such as *Bacteroidetes* and *Firmicutes* phyla, and an increase in bacteria with pro-inflammation activity, such as *Proteobacteria*, following alcohol consumption [47,48,49]. Unlike chronic alcohol consumption, binge drinking pattern (a frequent form of alcohol consumption, defined as 5 or more drinks for men and 4 or more drinks for women within 2 h) has not shown homogeneous results even using similar experimental designs. Some studies have found an effect of binge drinking on IMB (increased 16S rDNA levels) [53], but others have obtained negative results [54]; therefore, more studies are needed to elucidate this relationship.

By incompletely understood mechanisms, alcohol abuse leads to a disruption of the intestinal barrier integrity which in combination with the mucosal injury induced by alcohol, increases the permeability of the mucosa [55]. The intestinal barrier is a semipermeable structure that allows the uptake of essential nutrients and immune sensing while being restrictive against pathogenic molecules and bacteria [56]. It is composed of multiple layers of defense that included mucus with antimicrobial peptides and immunoglobulin A molecules, monolayer epithelial cells firmly join by tight junction proteins and the inner lamina propria where the immune cells reside and play an essential role in protecting the intestinal mucosa against invading bacteria [57]. Numerous studies have demonstrated that ethanol, its metabolites, and alterations of the gut microbiome suppress intestinal tight junction protein expression [58,59,60,61] producing that the epithelial layer becomes leaky or “permeable”. Alcohol increased gut permeability affects mucosal immunity and allows the translocation of bacterial or some critical components of their membrane into the bloodstream [47], reaching other organs that can be damaged. LPS (lipopolysaccharide), Gram-negative bacteria membrane main product, and other bacterial metabolites reach the liver via the portal vein where they are enabled to induce the activation of the inflammatory processes. A study in rats has shown that only two weeks of alcohol administration disrupts the intestinal barrier and after two weeks more, liver injury occurs [62]. In the liver, gut-derived molecules interact with the hepatocytes, parenchymal cells, and immune cells causing injuries including hepatic steatosis, hepatitis, fibrosis, cirrhosis, and hepatocellular carcinoma [63].

**Figure 2:**
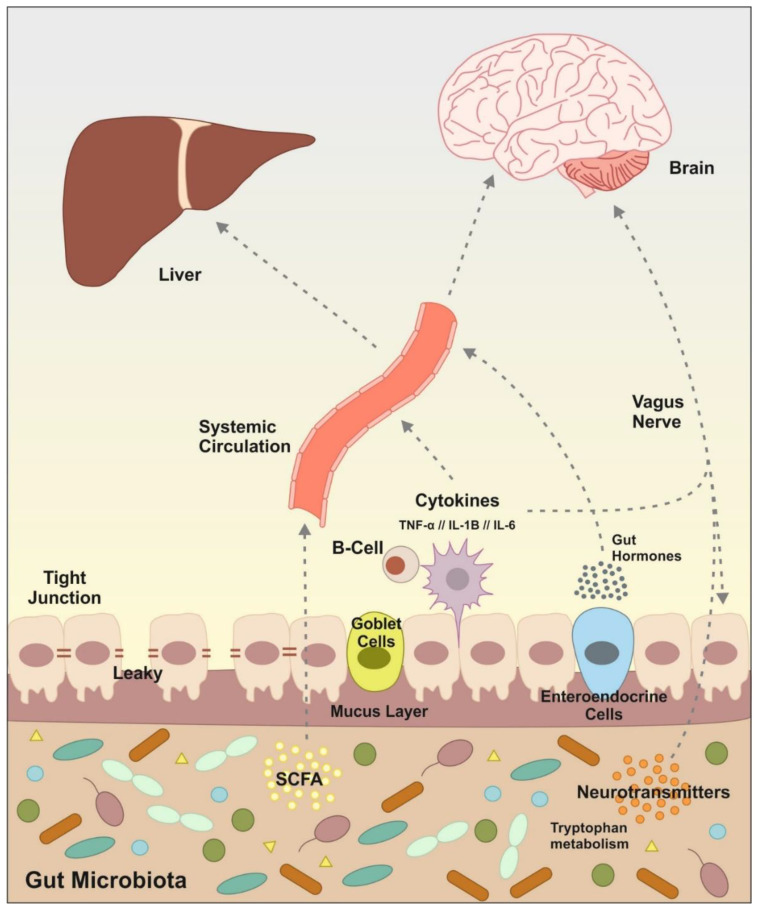
Principal signaling pathway and molecules involved in the communication microbiota/gut to the brain and liver. Gut microbiota can signal to the brain and liver through multiple direct and indirect mechanisms. Microbiota produces neurotransmitters, tryptophan metabolites, fermentation metabolic by-products such as short-chain fatty acids (SCFAs), the release of cytokines by immune cells and gut hormone signaling. Some of these molecules can activate the vagus nerve or reach the brain and liver via systemic circulation. Alcohol consumption causes dysregulation in the intestinal microbiota, which leads to an alteration in this communication and subsequently causes alterations in brain and liver functions.

The liver is not the only organ distant from the gut that has been associated with deleterious effects of intestinal dysbiosis due to alcohol. The brain is also a target of the gut microbiota. In recent years, there has been a growing awareness of the crosstalk between our intestinal bacteria, the central nervous system (CNS) and behavior [64] (Figure 2).

Numerous sources of evidence gathered from experiments carried out in rodents show that modifications in the composition of gut microbiota impact in the brain functions and behavioral aspects [65], including the predisposition to high alcohol consumption [66]. Leclercq et al. [67] found a correlation between leaky gut and inflammation with modifications in scores of depression, anxiety and social interactions in alcohol craving. Along the same line, it has been shown that rats replicate several behavioral and biochemical alterations after stool transplantation from patients with depression and anxiety behaviors [68]. In the study of Xiao et al. [52] transplanted microbiota in mice from alcoholic to healthy, developed emotional symptoms, such as anxiety, which occurs during abstinence.

The IMB maintains bidirectional interaction with critical parts of the CNS [68]. The microbiota–gut–brain axis communicate both organs not only through neuronal signals (neurotransmitters), it also depends on endocrine (hormones and gut peptides) and immune signals (cytokines), and microbiota derived metabolites (short-chain fatty acids -SCFAs-, branched chain aminoacids, and peptidoglycans) acting together to regulate host physiology and microbiota composition [64]. Gut microbiota are able to produce various of the aforementioned metabolites that act on enteroendocrine cells, the vagus nerve or by translocation throughout the gut epithelium into the systemic circulation and may have an impact on host physiology.

The vagus nerve is the fastest and most direct route that connects the gut and the brain, it is composed of afferent and efferent fibers [69]. This nerve transmits information from the gastrointestinal, respiratory and cardiovascular systems and gives feed-back to the visceras. Gut–brain signaling occurs primarily via the vagus nerve, vagal afferents sense intestinal molecules, e.g., intestinal hormones, neurotransmitters or bacterial by-products [64]. The alterations of the vagal activity at intestinal level are associated with bacterial overgrowth and bacterial translocation [70]. As observed by Freeman et al. [71] in alcohol withdrawal and during chronic alcohol feeding, there is a dysregulation in vagal signaling that could result in neuroinflammatory processes. 

The main products of the fermentation of dietary fiber, SCFAs (acetate, propionate and butyrate principally) are considered as one of the main direct or indirect mediators of microbiota–gut–brain interactions [72]. The highest production of SCFAs occurs in the proximal colon, where they are quickly and efficiently absorbed, since only 10% of the acids are excreted with the feces [73]. The rest of the SCFAs reach the circulatory system via the superior or inferior mesenteric vein, reaching the brain and crossing the blood–brain barrier thanks to monocarboxylate transporters thus being able to act as signaling molecules between the gut and the brain [74]. IMB metabolic activity can be modified due to chronic alcohol consumption. Specifically, chronic alcohol consumption could reduce the SCFAs count through the reduction in some *Firmicutes* genera, such as *Faecalibacterium* and *Ruminococcaceae*, on which the production of SCFAs depends [75,76]. Furthermore, it has been described that alcohol consumption would also have effects on other microbiota derived metabolites, leading to increases in branched-chain amino acids [77] and peptidoglycans [78]. However, studies showing the effect of alcohol on these microbiota derived metabolites are scarce.

Alcohol alters the composition of the IMB, resulting in an alteration of the amount and type of neuroactive substances produced by the microbiota, which may lead to behavioral alteration [79]. Gut–brain communication is disrupted by alcohol-related immune and gut dysfunction [80]. Alcohol modifies the intestinal microbiota, pH and permeability of the intestine, causing an increased entry of endotoxins into our CNS and brain, leading to neuroinflammatory processes.

## 3. Effects of Alcohol on Immune System: Putting All the Pieces Together

Traditionally, it has been described that alcohol acts on the immune system depending on several variables, including consumption pattern. Thus, several studies indicate that light to moderate consumption leads to reduced levels of systemic inflammation or improved responses to vaccines. In contrast, chronic heavy drinking (CHD) is often associated with a deficient immune response [15,81]. In this way, this consumption pattern is associated with an increased risk of infection by several viruses [82], and it has been suggested that it may lead to a greater severity and mortality from the recent COVID-19 pandemic [83,84,85]. In addition, subjects with Alcohol Use Disorders (AUD) show a worse postoperative recovery, a poor response to vaccination or a slower recovery from infections [81]. CHD alters innate and adaptive immune responses [82,86] and can affect a large number of systems through them, since this type of consumption has been associated with damage to different tissues such as pancreas, liver, gut, circulatory system or nervous system [87], and there are several studies that attribute, at least in part, a role of persistent systemic and local inflammation in these conditions [88].

**Figure 3:**
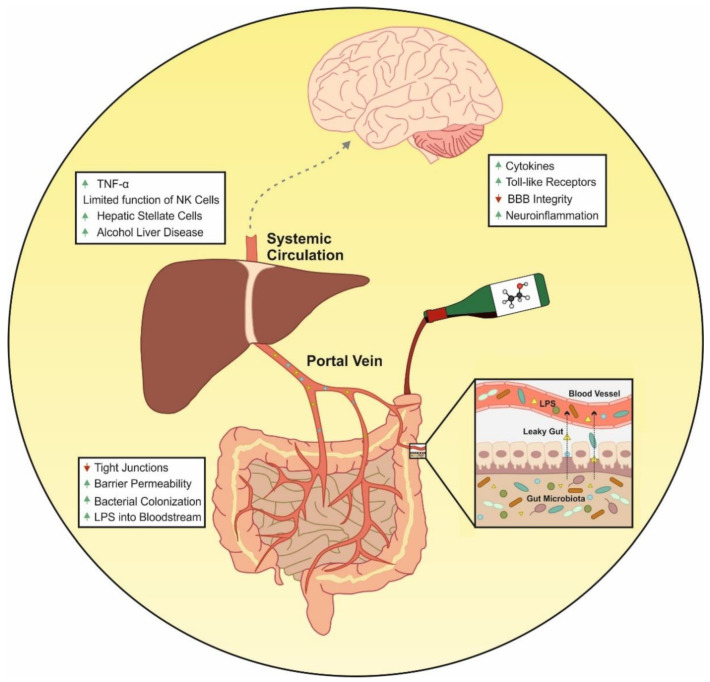
Effects of alcohol on the immune system. Alcohol consumption increases intestinal permeability through the suppression of intestinal tight junction protein expression. This alteration allows the translocation of bacterial products to the systemic circulation. The gut-derived bacterial components together with LPS activate the immune cells localized in the systemic circulation or in target organs such as liver and brain. This causes the increase in pro-inflammatory components that can lead to alcohol liver disease or increased states of neuroinflammation.

Some of the effects of CHD on cells of the immune system include reduction in T-cell numbers, loss of naïve T-cells, increased CD8+ T-cell activation and proliferation, or alterations in monocytes [81,89]. Together with the effect of alcohol consumption on Toll-like receptors [90,91,92], one of the most reported data are the upregulation of several cytokines after alcohol administration [93]. In fact, a recent meta-analysis [94] studied the differences in cytokine patterns presented by subjects with AUD and concluded that they show a higher concentration of cytokines than control patients. Furthermore, these authors found clear differences depending on the different stages of AUD illness: active drinking, withdrawal and various periods of abstinence. Such results are very interesting in order to develop potential biomarkers of alcohol consumption [95], as well as pharmacological alternatives to treat alcoholism [96]. Although the effect of alcohol on the immune system occurs at the systemic level and affects various organs, we will focus on the effect of this substance on the gut, brain and liver (Figure 3), due to the importance of these organs in the relationship between alcohol consumption, intestinal microbiota and the immune system [97].

The gut is the largest organ with immune function in our body [98] and, in order to regulate the immune response, the gut must keep the homeostasis of the intestinal barrier in check [99,100]. As mentioned above, alcohol consumption increases intestinal permeability through the suppression of intestinal tight junction protein expression. This alteration allows the translocation of bacterial products to the systemic circulation. The gut-derived bacterial components together with LPS activate the immune cells localized in the systemic circulation (peripheral blood mononuclear cells), or in target organs [101]. The release of LPS into the bloodstream results in the activation of two important targets of the immune response: TLR4 and nucleotide-binding domain leucine-rich repeat containing 3 (NLRP3) or cryopyrin. In that sense, research on the role of TLRs in the pathogenesis of alcoholism has revealed that these receptors mediate the development of a neuroinflammatory effect in the CNS derived from alcohol consumption [102,103].

The activity of these receptors triggers the activation of a number of molecular pathways that result in the expression of genes of the innate immune system, mainly proinflammatory factors, that contribute to a permanent neuroinflammatory state of the CNS. A study conducted in 2015 showed that blocking TLR4 function most of the neuroinflammatory effects produced by ethanol were diminished [104]. In another study, adolescent mice that consumed ethanol intermittently (3 g/kg) for two weeks, showed that this consumption pattern leads to an activation of TLR4 signaling pathways, an up-regulation of cytokines and proinflammatory mediators, in addition to synaptic and myelin alterations. TLR4-deficient mice prevented such neuroinflammation, synaptic and myelin alterations, as well as long-term cognitive alterations [105].

Interestingly, in addition to supporting neuroinflammation, TLR signaling is likely engaged in the mechanisms of regulation of the functional activity of neurotransmitter systems, which may contribute to the formation of a pathological demand for alcohol [106]. Together with TLRs activation, the production of cytokines, which can cross the blood–brain barrier (BBB), have harmful effects at CNS level [102]. To that respect, the BBB is known to be a major target for alcohol. Long-term consumption produces serious impairments in the BBB permeability and integrity since alcohol inhibits the expression of BBB structural and functional proteins, promoting inflammation and oxidative stress [107].

The immune response, therefore, would be one of the main channels through which the gut-brain axis establishes communication [108]. Since alcohol is responsible for inducing changes in this communication, leading to peripheral and central inflammation [109], dysfunction in gut microbiota and the subsequent affection of the immune system is linked to the development of mental illnesses, brain dysfunction and neurodegenerative disorders like Alzheimer’s and Parkinson’s diseases [110,111,112,113]. Interestingly, central neuroinflammation is maintained after cessation of alcohol consumption, compared to peripheral activation [114] and during periods of abstinence [108]. Finally, in relation to the effect of alcohol on neuroinflammation, a study by Lowe et al. showed an attenuation of alcohol-induced neuroinflammation after reducing the gut bacterial load, as a result of antibiotic treatment [115]. We could hypothesize that by reducing the gut bacterial load, lower amounts of bacterial components would reach the systemic circulation, leading to reduced activation of pro-inflammatory components.

In addition to the central inflammatory effect, CHD induces a peripheral inflammatory response that plays an important role in the development of alcoholic liver disease (ALD) [108]. ALD is a broad term that refers to a variety of liver ailments. In particular, numerous clinical and experimental research [116,117,118,119,120] have revealed the role of immunology in fueling inflammation and progression of ALD. As said before, alcohol consumption modifies the barrier function of the intestinal mucosa, leading to an increased bacterial load together with high levels of LPS that enters the portal circulation through alcohol-disrupted barrier of gut. LPS activates innate immunity via TLRs expressed by immune cells producing immunological challenges that disrupt the liver’s finely tuned immune pathways [121,122,123]. Some other cellular sensors of pathogen- or damage-associated molecular patterns (PAMPs/DAMPs) are further activated, leading to the generation of pro-inflammatory cytokines like TNF-α and ILs, which contributes to ALD [123]. Both innate and adaptive immunity are known to have a role in the pathogenesis of ALD [124]. As a result of continued alcohol misuse, alcoholic hepatitis and fibrosis develop. At this point, the oxidative breakdown of alcohol limits the function of immune cells like natural killer (NK) cells, which cause activated hepatic stellate cells (HSCs) to enter in apoptosis, resulting in mild fibrosis [125,126,127]. Finally, fibrotic distortion of tissues and blood vessels, as well as cell necrosis, characterize the ultimate stage of ALD. The failure of the liver to eliminate microbial and other circulating pro-inflammatory chemicals, as well as the release of immunogenic cellular debris from necrotic hepatocytes, results in prolonged immune system activation and worsens the condition [128,129].

## 4. Conclusions

Chronic excessive alcohol consumption causes inflammation in a variety of organs, including the gut, brain and liver. While alcohol has direct effects on the gastrointestinal tract when it comes into touch with the mucosa, the majority of alcohol’s biological effects are due to its systemic dispersion and delivery through the blood. Alcohol has been proven to affect the microbiome in the gastrointestinal tract, with alcoholics having a different and higher bacterial load in their gut. Once the integrity of the gut mucosa is impaired, LPS enters the portal circulation contributing to enhance the inflammatory changes in other organs such liver and brain.

## Data Availability

Not applicable.
